# Walking together: exploring australian parent experiences of walking in nature with their preschool children with autism using a bioecological lens

**DOI:** 10.1080/17482631.2026.2719072

**Published:** 2026-08-15

**Authors:** Carolyn Galbraith, Tonia Gray, Marion Sturges

**Affiliations:** a Centre for Educational Research School of Education, Penrith Campus Western Sydney University, Penrith, Australia; b Centre for Educational Research School of Education, and the World Leisure Centre Excellence (WLCE), Penrith Campus Western Sydney University, Penrith, Australia

**Keywords:** Bioecological, children with autism, developmental growth, nature, outdoors, parents of children with autism, walking

## Abstract

**Background:**

Walking regularly in natural settings is a common healthy activity for Australian parents and their preschool children. Extant research conveys that walking in nature provides cognitive, emotional and physical benefits for both parents and children. Children with autism are less likely to participate in physical activity or access nature regularly, and autistic adults are less likely to hold pro-environmental views. While research exploring the experiences of typical parents and preschoolers exists, the perceptions of parents of preschool children with autism on walking is yet to be studied.

**Methods:**

This paper examines the experiences of parents of children with autism using Bronfenbrenner’s bioecological model as a theoretical framework. We recruited 20 parents of preschoolers with autism and a comparison group of 12 parents of preschoolers without autism and invited them to complete an online qualitative survey about walking together in nature.

**Results:**

Five themes were generated from their responses, including *calm*, *connection*, *coordination* and *communication*, with an additional theme of *priority*. Parents identified that walking in nature provided opportunities for developmental growth for their children such as increased communication, connection and calm, but also highlighted challenges such as differences in moving together in coordination.

**Conclusion:**

We advocate that supporting family priorities includes supporting their ability to access everyday activities such as walking in nature, a common activity that provides rich opportunities for parents and their preschoolers with autism.

Walking in natural settings with others is an enjoyable, healthy activity and is one of the most common recreational activities undertaken by Australians (Australian Institute of Health & Welfare, 2024; Horton et al., 2014). It has been described as a foundational life activity for developing relationships (Donald, 2021), adding a sense of meaning to daily life (Wensley & Slade, 2012) and combining everyday movement with play (Clement & Waitt, 2017). Walking in natural rather than urban settings has been posited as even more beneficial, improving memory and attention, mood, and physiological stress (Boere et al., 2023; Marselle et al., 2013). Research on parent perspectives of walking with their children has found that parents enjoy walking with their children more than by themselves (Filanowski & Slade, 2023). Correspondingly, regular parent and child walking improves adult wellbeing (Gustafsson et al., 2021) and creates stronger family connections (Izenstark & Ebata, 2017; Izenstark et al., 2021). Children benefit in myriad ways by accessing nature, with cognitive, socio-emotional and physical advantages associated with regular time in natural settings (Chawla, 2015; Dowdell et al., 2011; Harper et al., 2026; Liu et al., 2025; Siatou et al., 2024). These affordances may extend to children with autism.^1^ Although limited, existing research reports that exposure to nature for children with autism is associated with improvements in physical fitness, concentration, mood and social interaction, as well as deep enjoyment (Fahy et al., 2021; Galbraith & Lancaster, 2020; Li et al., 2019).

While research examining the walking experiences of typical parents and preschoolers exists (Clement & Waitt, 2017), the perceptions of parents of preschool children with autism on walking is yet to be studied. This is indicative of a significant research gap, as children with autism are less likely to access nature, and autistic adults exhibit fewer pro-environmental attitudes than others due to a combination of structural and systematic barriers, which may include lack of support for parents. (Huang & Kang, 2021; Kanamori et al., 2024; Taylor et al., 2021). If children with autism walk in nature less frequently than other children, they are potentially less likely to reap the benefits that this offers. Supporting the families of preschoolers with autism to increase time walking in nature must begin by considering their lived experiences.

Our paper will begin by first outlining the theoretical perspective of Bronfenbrenner’s bioecological model (Bronfenbrenner & Morris, 1998; Bronfenbrenner, 1979), followed by a brief discussion of the broader literature focusing on parents of preschoolers with autism and the natural settings that families access. The selected methodology will be described, and the results of the review presented thematically, before a subsequent discussion of the ideas generated and the identified gaps in the literature.

## Bronfenbrenner’s bioecological model

**Figure 1.**
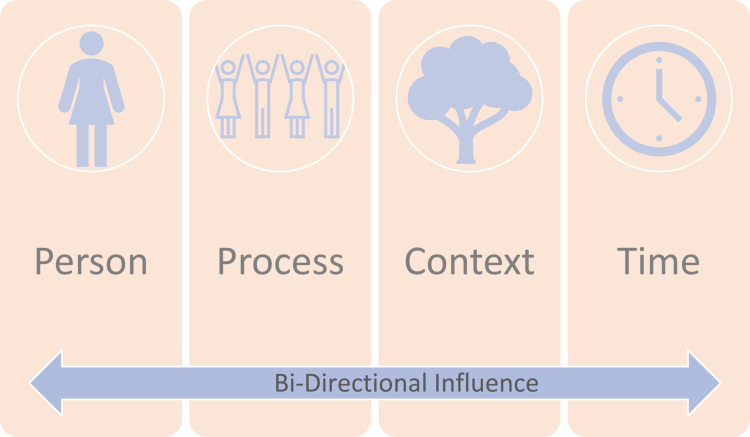
Bronfenbrenner’s bioecological (PPCT) model.

This paper examines the experiences of parents of children with autism using Bronfenbrenner’s bioecological model (Bronfenbrenner & Morris, 1998; Bronfenbrenner, 1979) as a theoretical framework (see Figure 1). Situating research within theoretical frameworks can provide a deeper layer of understanding and can ensure that the concepts examined are relevant to the larger discussions in the field (Murphy, 2020). The ecological, later bio-ecological framework or PPCT (Person-Process-Context-Time) was introduced as a conceptual model by Bronfenbrenner within the field of developmental psychology to explore the way humans develop based on their environment, from microsystems such as family to the macrosystem of the wider culture (Bronfenbrenner & Morris, 1998; Bronfenbrenner, 1979). It has been used as a framework to explore the relationship between nature and children (MacQuarrie et al., 2022; Pryor et al., 2005) and for research with children with autism (Brisendine et al., 2021).

The PPCT model suggests that factors inherent within the person themselves (individual factors), within active interactions in their immediate surroundings (the process) and larger systems (the context) affect one another in a bidirectional way. The amount of time also has a large effect, with regular processes more influential than one-off events (Bronfenbrenner & Morris, 1998).

Bronfenbrenner’s bioecological framework explores the way regular, active participation across different settings affects developmental growth (Bronfenbrenner & Morris, 1998). While some researchers have argued that Bronfenbrenner has decreased relevance in studies of nature due to its anthropocentric focus (Elliott & Davis, 2020), Bronfenbrenner’s work does acknowledge the importance of the physical environment, and the effect of natural biodiversity. He defined settings as actual tangible locations, and his initial works explored the differences that institutional settings and family homes could have on interactions and developmental growth. Bronfenbrenner himself was influenced by the wooded landscape setting he was raised in, whose biodiversity inspired his ideas about ecology (Bronfenbrenner, 1979).

## Benefits of walking in nature

Walking in nature with others involves active participation in a setting which can provide increasingly complex challenges, from following pathways to responding to unexpected wildlife and changes in weather (Vella-Brodrick & Gilowska, 2022). Chawla (2015), who reviewed the evidence on the effect of nature on children’s development reported advantages across areas including physical health and fitness, emotional and cognitive processing, affiliation and play, and connection to the environment. More recent research has observed types of developmental growth associated with accessing nature include richer communication (Novikova et al., 2024), higher intelligence (Liu et al., 2025), enhanced socio-emotional and academic outcomes (Mann et al., 2022), and measurable differences in attention and working memory (Vella-Brodrick & Gilowska, 2022). Walking in natural settings may strengthen family relationships (Izenstark & Ebata, 2017; Izenstark et al., 2021) and add a sense of fun and playfulness to daily exercise (Clement & Waitt, 2017).

These benefits may be available to children with autism, with research associating access to nature with increased social interactions, deeper concentration, improved physical fitness and mood, as well as opportunities to spend time on specific interests (Fahy et al., 2021; Galbraith & Lancaster, 2020; Li et al., 2019). We were unable to find any research exploring family walking with children with autism, or any large-scale experimental studies that have compared nature interventions for children with autism to indoor occupations. However, Fan et al. (2023) reviewed 24 small-scale studies, including 717 participants, involving children aged between 5 and 18, involving outdoor recreation therapy. While the therapies were heterogeneous, and the risk of bias remained high in almost half the studies, the researchers considered evidence remained of positive health and wellbeing outcomes associated with nature-based therapies (Fan et al., 2023).

While the research on children with autism and nature is scant, the literature focusing on preschool-aged children with autism is even more limited (Huang & Kang, 2021). Davis (2009, *p*. 227) claimed that there is a ‘hole’ in current research because the early childhood years are often missing in literature exploring sustainability, and Syrjämäki et al. (2023) argued that children with autism in the early childhood education sphere were an under researched population. However, the early childhood years may be vital due to the long-term patterns of family interactions which develop over this period (Gerstein & Crnic, 2018). While time outside benefits all children, preschoolers with autism may have particularly unique needs associated with accessing nature which are currently under-acknowledged.

## Insights from parents

Drawing on the perspectives of parents of children with autism may challenge assumptions about the strengths and needs of children with autism, including those associated with time in nature. Respecting parent perspectives as those of members and partners, rather than simply caregivers, is essential to accurately explore the complexities within the autism community (Smith et al., 2023). Parents of children with autism are in a unique position to observe their children’s development over time and are often involved in long-term advocacy (Boshoff et al., 2017). Researchers have found parent views of their children with autism to be accurate and insightful (Coonrod & Stone, 2004; Harte, 2009; Locke & Mitchell, 2016; Rabba et al., 2019). A review of family-focused autism research found that it is still a relatively underdeveloped area but has a high potential to influence clinical services (Cridland et al., 2015). Providing space for parent voices, therefore, is essential to more accurately report on time in nature for children. However, to date we have located no research exploring parent perspectives on walking with their preschool children with autism in natural settings. This impelled us to ask: what insights do parents of preschool children with autism have about walking together in nature, and further, in what ways do parents of preschool children with autism feel that walking with their child in nature is a priority?

## Methodology

A qualitative, interpretive methodology was selected as the most appropriate way to gain insight into the lived experiences of parents raising young children due to the way it examines reality through the constructed experiences of individuals (Ayton et al., 2023). The *Walking Together* project was designed to be consistent with the axiological values of an interpretive paradigm, where reflection is essential at each stage. In this project, recruitment, data collection and data analysis were thoroughly documented, in order to enhance transparency and dependability. As part of the larger project, participants were invited to respond to questions about a connectedness to nature index, as referenced in Galbraith et al. (under review), a question about supports needed (Galbraith et al., under review), and open-ended questions about walking with their child in nature, which will be described in this paper. The *Walking Together* project was approved by the Western Sydney University Human Ethics committee (H16313) in accordance with NHMRC guidelines. The survey was open for eight weeks, between April and May 2025.

### Recruitment

Participants were recruited using existing Australian Facebook groups as documented in our earlier works (Galbraith et al., under review). Research suggests that recruiting from existing groups may be more effective for less researched samples (Benoit et al., 2005; Ellard-Grey et al., 2015), and the use of Facebook may reduce time and cost compared with traditional methods (Whitaker et al., 2017). Participants recruited for this research included parents of preschool-aged children with autism, defined as between two and five years, and parents of preschool-aged children without autism. In order to centre the perspectives of parents, it was essential to use a method designed to reduce assumptions about what may be unique about a selected group. The use of qualitative comparison groups, where participants with and without a particular experience share their responses may reduce assumptions about what is unique to that group (Lindsay, 2018). In this study, the administrators of 12 Facebook support groups focusing on parenting in general or parenting a child with autism were contacted, with six of those administrators providing permission to post details of the study. The study aimed for around 12–15 participants in each group. In reflexive thematic analysis, the researcher’s perception of data richness guides their choices on when to cease data collection; other research in qualitative data collection has found that a sample size of 12 is adequate to represent diversity in data (Braun & Clarke, 2021; Hennink & Kaiser, 2022). Over 60 surveys were sent out initially, with the survey completed in full by 12 parents of children without autism, and 20 parents of children with autism (see Table I). Respondents were asked for their postcodes, ethnicity and parenting role and children’s age. Participants from five of the eight states and territories were represented, with a quarter of respondents from each of the groups of participants indicating that they were from culturally and linguistically diverse communities including Aboriginal, Indian, Sri Lankan, and Maltese. All children were aged between two and five, and two participants identified as fathers, with the remaining participants identifying as mothers.

**Table I.** Participant characteristics.

Participant no.	Parent role	Child diagnosis	Type of prompt
S1	Mother	Autism	Specific walk
S2	Mother	Autism	Specific walk
S3	Mother	Autism	Walking in general
S4	Father	Autism	Walking in general
S5	Mother	Autism	Walking in general
S6	Mother	Autism	Walking in general
S7	Father	Autism	Walking in general
S8	Mother	Autism	Specific walk
S9	Mother	Autism	Walking in general
S10	Mother	Autism	Specific walk
S11	Mother	Autism	Specific walk
S12	Mother	Autism	Walking in general
S13	Mother	Autism	Walking in general
S14	Mother	Autism	Walking in general
S15	Mother	Autism	Walking in general
S16	Mother	Autism	Specific walk
S17	Mother	Autism	Specific walk
S18	Mother	Autism	Specific walk
S19	Mother	Autism	Specific walk
S20	Mother	Autism	Specific walk
R1	Mother	Without Autism	Walking in general
R2	Mother	Without Autism	Walking in general
R3	Mother	Without Autism	Walking in general
R4	Mother	Without Autism	Walking in general
R5	Mother	Without Autism	Walking in general
R6	Mother	Without Autism	Walking in general
R7	Mother	Without Autism	Walking in general
R8	Mother	Without Autism	Walking in general
R9	Mother	Without Autism	Walking in general
R10	Mother	Without Autism	Walking in general
R11	Father	Without Autism	Walking in general
R12	Mother	Without Autism	Walking in general

### Data collection

In our study, participants were given a link to a webpage which described the study and then provided a private link to the online survey. The use of online qualitative surveys was selected as an important way to ensure parent voices occupied a central place in this research. Thomas et al. (2024) suggest that these qualitative surveys offer a rich potential for researchers who want to reduce barriers for participants and ensure their subjective experience, rather than that of the researcher, remains paramount. Braun and Clarke (2021) suggested that online qualitative surveys were a useful and yet underused method of data collection. In our study, the online surveys were hosted by REDcap, a secure, web-based application designed exclusively to support data capture for researchers and hosted and supported by the Women and Children’s Health Research Institute at the University of Alberta (Harris et al., 2009). Participants were given detailed information about the study before providing written consent by signing online. Respondents were informed about the numbers of helplines in the event that the questions evoked challenging emotions and were offered a $25AUD gift card in return for their time.

In order to provide richer data, half the participants who had children with autism were invited to write about their experiences immediately following a walk outdoors. The other half of the parents who had children with autism, and the parents of children without autism, were asked to respond to questions without the prompt to walk. Research has found that around 23 minutes is considered ‘walking distance’, while as little as 10 to 20 minutes in nature is enough to positively impact the health of walkers (Hook, 2024; Meredith et al., 2020). Providing multiple ways of understanding through different processes, such as writing from memory or writing directly after walking, may enhance data richness (Golafshani, 2015).

### Data analysis

**Figure 2.**
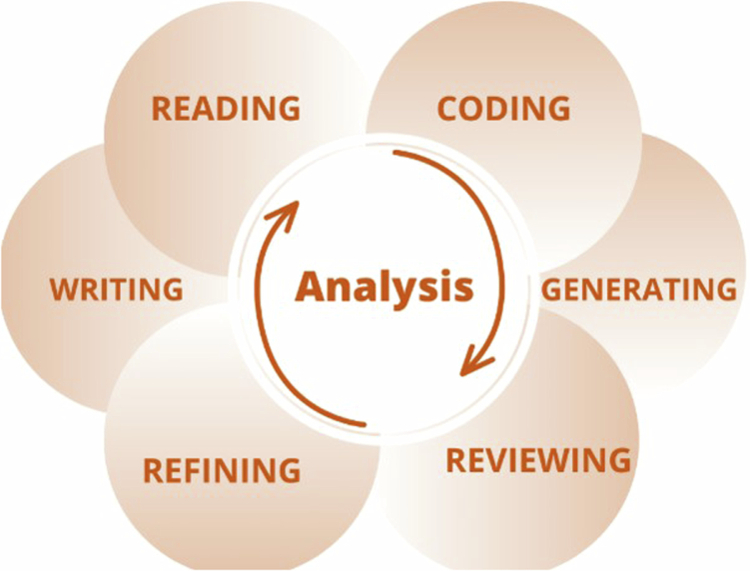
Six phases of reflexive thematic analysis.

Data were analysed using reflexive thematic analysis, where repeated patterns of ideas are interpreted using thematic coding (Braun & Clarke, 2006). The six phases of thematic analysis were undertaken reflectively, with responses read multiple times for familiarisation of the data, followed by coding, generation of initial themes and the process of reviewing, refining themes and writing to contextualise the analysis (Braun & Clarke, 2021) (see Figure 2). Coding was done by hand, with no computer programme utilised; an excel workbook was created and then used to order ideas and phrases to generate initial codes and later themes. Multiple coders were not used in this study, as interpretation rather an objective consensus is the aim of reflexive analysis, but the outcome of analysis was reviewed by the coauthors, and any disagreements resolved through discussion. Analysis was also guided by the overall framework of Bronfenbrenner’s PPCT model (Bronfenbrenner & Morris, 1998). Participant responses were considered in relation to Bronfenbrenner’s ideas about developmental growth occurring through bi-directional interactions with others and the environment itself.

## Results

Participants responded to the open-ended qualitative questions with thoughtful and diverse responses. Parents of children with autism provided 20 relevant responses (coded S for sample), and parents of children without autism provided 12 relevant responses (coded R for response). Half the parents of children with autism, and all the parents of children without autism were asked about their experiences walking in nature generally and in what way walking in nature was a priority for them. The survey questions they were asked to respond used the following prompts:

*Can you tell me about walking with your child in nature, such as bushlands, creek or beach? Feel free to write as much as you like.*

*Do you feel that walking with your child in nature is a priority—could you explain why or why not?*

The final group, ten of the parents of children with autism, were invited to take their child on a 20-minute walk in nature and to write about it immediately afterwards. Their prompt included the following:

*Please go with your child on 20 minute walk in nature, and then write about it afterwards. Feel free to write anything you like about your experiences. There are no right or wrong answers.* T*o make it easier, bring this survey on your phone or other device and complete it immediately after walking with your child. You could write about the space in nature you chose and why you chose it - is it a new place or a place you’ve been before? Does your child stay with you while walking, move ahead, linger behind? Do you need to hold on or does your child choose to hold onto your hand? Think about the way your bodies are moving together. Communication - this might be talking, looking, pulling at your hand, making sounds or another way of sharing a message. What communication did you notice? What was your child interested in and how did they show it? Did your child show they remembered anything from last time they walked, and how did they show it? Was there anything that could have made the experience easier for you?*

Five themes were generated from their responses, including *calm*, *connection*, *coordination* and *communication,* along with a final theme, *priority*. The results from these themes are presented below.

### Calm

Nature was considered a place of peace and relaxation by respondents who had preschoolers with autism. The presence of trees and water, and the possibilities inherent in a biodiverse landscape, evoked a sense of serenity.

*She is much calmer in nature, near water, trees and animals. It's very regulating.* (S12).

One participant felt that the lack of other people allowed their children to relax.

*They love to walk in nature. I feel it calms them somewhat, particularly if there aren't any other people (public) present. They are free to be themselves and there are less eyes for judgement than when at a playground.* (S6)

Another participant felt the reduction of sensory stressors increased calm.

*It is [removed] from sensory overload and provides a calm quiet environment that is therapeutic to his busy brain*. (S14)

Parents who had preschoolers without autism responded similarly, noting that outdoors provided necessary calm.

*I find walking with my child outdoors in nature very calming. If she is having a hard day I can take her outdoors and this will help her find peace in herself.* (R6)

### Connection

Connecting the individual to a wider world, including other people, the landscape, and specific interests was a repeated idea respondents offered. One parent of a preschooler with autism commented that walking in nature provided time to reflect on their parenting journey.

*I often go for bush walks with my children, including my autistic preschooler...When walking I often reflect on how far he has come in a short period of time in regards to language and regulation and our walks demonstrate this so well, and it's always been a nice way to connect with each other and with nature.* (S1)

Parents of children without autism were more likely to use words such as ‘family’ or ‘bonding’ when talking about their time outside, and to focus on the activity creating a sense of who they were as a family.

*We reduce stress and promote a sense of family and wellbeing.* (R1)

*Going for a walk with my child in nature not only is an incredible bonding time for us both. But it's creating a healthy habit and it's also made me as the mother slow down and walk slower and notice more on my Walks. It's an incredible benefit to both our emotional and mental wellbeing.* (R9)

Parents of children with autism were more likely to talk about the way being in nature helped their child connect to themselves and their own emotions, and the way this gave insight to themselves as parents about their child’s interests.

*My child feels very comfortable and excited to be around nature. He tends to notice details in nature that I do not notice. He also loves being near water as he finds it very calming. Swimming in the ocean or lake helps him to self regulate and feel calm.* (S14)

Both groups of participants mentioned connection to the land and water around them, with vivid descriptions of the birds, plants and sea creatures they came across. Those who responded directly after a walk were more likely to write in detail about the flora and fauna they encountered.

*He pointed out the grass length how wet it is as its been raining he points out the bugs moving we saw a cricket and flies ... we also heard and saw an eagle that has a nest in one of the tall trees.* (S20)

Connection to nature was a strong motivator to walk regularly for both groups of participants.

*In the longer term I want him to grow to appreciate and feel a connection with the land and nature, and to prioritise care for the environment and sustainability.* (R8)

### Coordination

Walking together in the same direction, at the same pace, involves coordination between family members. Parents of preschoolers with autism were more likely to mention the way they and their child moved together, referring to their children running off, walking ahead or behind, and either choosing or being reluctant to hold hands. In some cases, running off invokes stress in the parent, as their unpredictable movements can lead to danger.

*My child sometimes lingers behind, sometimes walks with me and sometimes walks ahead with his brother on the wider tracks.* (S1)

*I need to hold his hand the whole time (he’s usually unwilling to do this) and have 24/7 watch on him.* (S9)

*I am on guard and high alert because they can dart off at any moment or touch something that could hurt them or fall on uneven ground. They often refuse to hold my hand so I need to be ready to catch them.* (R2)

Other comments identified that running and jumping indicated joy and immersion in the natural environment. While it isn’t uncommon for young children to run ahead impulsively, consistent difficulty with staying near an adult can be stressful. Research has found that when walkers synchronise their movements, they also feel ‘in sync’ and connected, and this encourages pro-social, cooperative behaviour (Hu et al., 2022).

*When we are on the walk he will hold my hand occasionally and will point to birds which may appear and reach out to touch some plants which he knows. It is usually a quite enjoyable experience and very relaxing to go for a bushwalk with him.* (S7)

Children with autism are less likely to synchronise successfully with others, which can lead to challenges with feeling connected while walking (Carnevali et al., 2024).

*The situation can change immediately with her going too deep into water, running off or touching things that are dangerous. She doesn't know her limits and thinks she can swim when she can't. It's honestly more stressful than I can manage most of the time.* (S12)

### Communication

The early childhood period is one of immense change in language expression, with control over facial expressions, gestures, words and discussions increasing over time. Spending time in nature provided the opportunity to focus on children’s developing communication skills.

*He asked me questions about sea animals. We talked a bit about the shells and rocks we'd see, picked them up and inspected them, keeping a few in his pocket.* (S11)

Parents of children with autism were more likely to mention talking, pointing and asking questions while walking. Many of those responding referred to the way they talk about the animals, birds and plants that surround them while they walk together, and any changes which had occurred since last walking.

*Now that he is verbal, he asks a lot of questions about what things are, points out what has changed in the bush (like a fallen tree or flowers blooming) and asks about sounds he hears.* (S1)

A review of the literature focusing on outdoor communication suggested that open, natural spaces provide a setting that is supportive for different kinds of children’s voices, including louder and softer, curious and descriptive, fluent and dysfluent (Richardson et al., 2024).

*When I walk with her she'll ask a lot of questions about her surroundings, why plants are a certain colour, why are the ants walking in a line. It's a great learning opportunity and sparks curiosity.* (R7)

Researchers such as Bradshaw et al. (2021) have reported that autistic individuals present with a wide range of differences in social communication from infancy onwards. These differences may include delay in or absence of social smiling, gesturing, use of eye gaze to share mental states, offering objects, and vocalisations. Continuing difficulties in social communication is one of the chief concerns of parents of children with autism (Viljoen et al., 2020). However, parents reported that being in nature allowed for the possibilities of different kinds of communication.

*I asked her do u like trees around she nodded and do you want to go for more walk and she was happy.* (S18)

### Is walking a priority?

Further to the invitation to share experiences walking in nature was a question examining to what extent walking in nature was considered a priority by parents. Twelve parents of preschoolers without autism, and ten parents of preschoolers with autism, responded to the question: “Do you feel that walking with your child in nature is a priority—could you explain why or why not?”.

After responding to a question about their walking experiences, many respondents replied that they considered walking in nature to be a priority. Parents of preschoolers without autism offered the following comments:

*Yes, my children's attitude completely changes. Once we go outside. They are happier and I love to explore.* (R10)

Parents cited the effect of the natural setting on individual growth, including stress reduction, physical activity, and improved mood, as reasons why walking in nature was considered a priority.

*Yes, it is as it will help her to get fresh air and make her appreciate nature.* (R11)

Parents of preschoolers with autism offered detailed reasons why walking in nature was considered a priority.

*Yes, it seems to reset her if she's been having a hard time.* (S13)

*Yes - he enjoys being outside and it regulates him well.* (S9)

Participants responded by explaining how the natural setting affected individual factors including reducing sensory overload, reducing stress and increasing enjoyment.

Three of the parents of preschoolers without autism, and two of the parents of preschoolers with autism, responded that walking in nature was not a priority.

*It is not a priority. With full time work and running a household with other children the priority is getting through the day, breakfast, school drop, work, shops, home for dinner, bath bed and then do it again. A walk is something we can do when the other stuff has been completed.* (R2)

*Unfortunately, it's not a priority as the effort to get her to go anywhere and everything that's needed for the trip, during the trip and the effort to get her out and to leave as well as manage the aftermath is too hard for one person.* (S12)

The responses from participants without children with autism speak to competing priorities within their lived experiences as parents. Walking in nature is considered an aspect of life that may be beneficial but not as beneficial as completing other tasks. Participant R2 lists the competing priorities of employment and household care.

Parents who had children with autism responded with individual factors such as the child’s walking stamina, the child’s ability to follow instructions before, during and after the walk, and the child’s personal needs to explain why walking regularly was not prioritised.

Parents of preschoolers with autism also responded equivocally with ‘yes and no’.

*Yes and no. The idea is but actually getting around to it is another story.* (S5)

*All these activities [therapy, preschool] take a lot of time so depending on when we can fit in a bushwalk, [it] can sometimes be a priority but often something that will be left out.* (S7)

*Yes and no. It can be hard to walk with my child as he is a twin and it can be hard to watch both.* (S8)

## Discussion

Bronfenbrenner’s bioecological framework (Bronfenbrenner & Morris, 1998; Bronfenbrenner, 1979) explores the opportunities for developmental growth which exist in the individual (biological factors) and the environment that they live in (ecological factors). The central claim of Bronfenbrenner’s framework is that active participation in regular interactions within settings affect development (Bronfenbrenner & Ceci, 1994). Walking in nature with others involves interactions between walkers, between other people who may also be outside, and with the natural setting itself. Natural landscapes are both complex and unpredictable settings which can affect developmental growth through physical, emotional and cognitive appraisals (Harding, 2008).

One key ontological question is to consider to what extent parent perceptions reflect the systems that Bronfenbrenner describes. The assumption at the heart of this exploration is that the unique perspective that parents of preschool children with autism hold is both accurate and important. In our study, participants made keen observations about the interactions between their child and the setting, as well as their child and other people. The experiences that they described can be organised into the four themes of *calm*, *connection*, *coordination* and *communication*. In accordance with Braun and Clarke’s methodology (2006), we acknowledge that these themes are generated from our own reflexive analysis of the data, the result of our individual histories, experiences and worldviews.

### Calm

**Figure 3.**
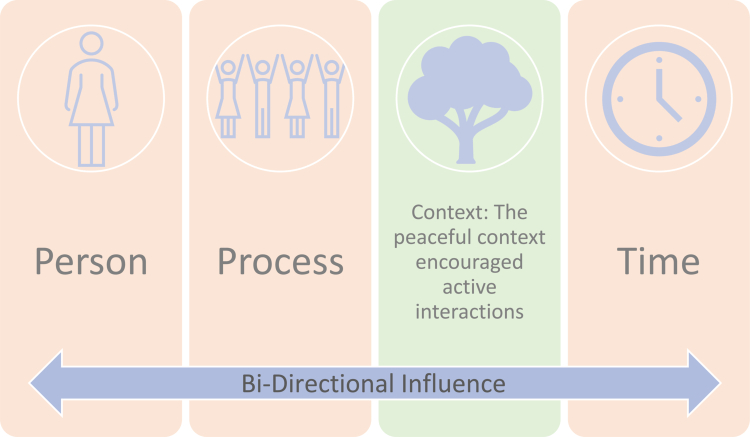
Importance of a calm context.

Participants reported that being in natural settings evoked a sense of peace in their child, and often themselves as well. Bronfenbrenner suggested that a chaotic environment, rather than a calm one, could be detrimental to developmental growth (Bronfenbrenner, 1979), hence the benefits of spending time in a peaceful setting. The presence of a green and blue landscape in natural settings may be inherently calming (Völker & Kistemann, 2015). The reduction of stressors such as the gaze of strangers or sensory disturbances such as noise and bright lights may allow children with autism to interact with others, take risks, and challenge themselves outdoors more freely, therefore encouraging active interactions and facilitating developmental growth (Blake et al., 2018). From a bioecological perspective, the peaceful context was essential for promoting the active interactions which encourage developmental growth (see Figure 3).

### Connection

Connection to others, to themselves, and to nature itself was an important aspect of walking in natural settings for participants who had children with or without autism. Parents of children without autism were more likely to mention a sense of family bonding, while parents of children with autism commented on their child’s connection to their own emotions and interests. Research has found that the deep interests of autistic individuals can serve as a bridge, a way for others to understand them as well as a way they can restore their own emotions when exhausted by social contact (Lizon et al., 2024). Walking itself can assist with deep, creative thought which may support children’s deep interests (Keinänen, 2016; Mäkelä & Aktaş, 2023). Equally, overt signs of bonding and connection, such as joint attention and experience-sharing language, may be less common in children with autism (Prelock & Nelson, 2012).

Both sets of participants emphasised the connection to nature that walking outside promoted. Positive childhood nature experiences are associated with a higher connection to nature in adulthood (Barrable et al., 2024). Supporting early, regular connection to nature in childhood may have both immediate and long-term benefits for children, increasing sustainable behaviours and perceived happiness (Barrera-Hernández et al., 2020).

**Figure 4.**
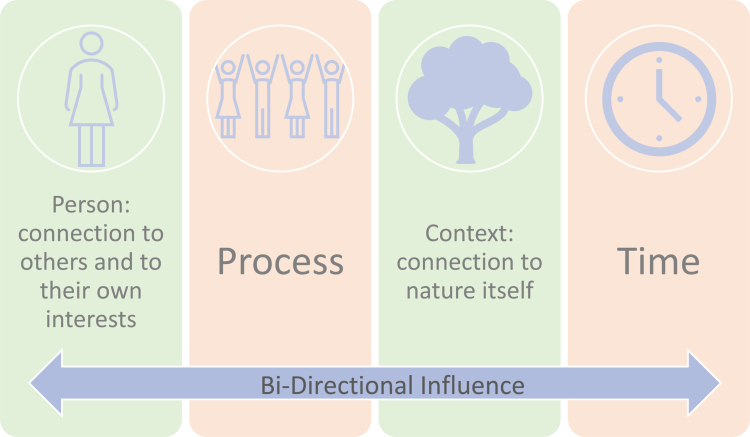
Connection between people and context.

Bronfenbrenner emphasised the bi-directional nature of developmental growth; the child influences their environment, just as the environment influences the child (Bronfenbrenner, 1979). Connection is formed between parent and child, and parent-child-nature, because of the setting, and the way the child’s actions are perceived by the parent. In this case, the setting provides opportunities for shared enjoyment, connecting parent and child, and connecting them both to the natural context in which they walk (see Figure 4).

### Coordination

**Figure 5.**
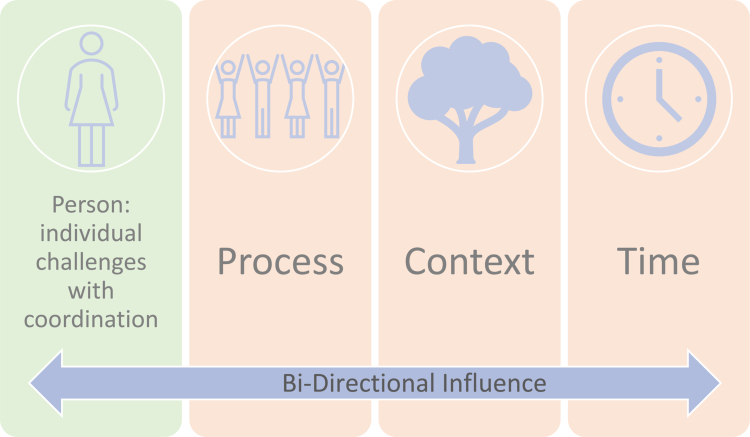
The challenge of coordination between people.

Differences in moving together while walking were noted by respondents, especially those who had preschoolers with autism, where running off was mentioned frequently. Exploring this through Bronfenbrenner’s lens, we can see the way personal factors, such as difficulty with synchrony, can affect the process of walking together, which may limit the pro-social benefits of walking together in nature (see Figure 5). If one walker finds the process stressful due to the need for constant vigilance, they may be less likely to repeat the experience. The literature suggests that not only do children with autism spend less time in nature than other children, but as adults, they may have fewer pro-environmental attitudes than others (Taylor et al., 2021). However, the research also suggests that skills in synchrony can be encouraged and improved through targeted intervention (Galbraith et al., 2026; Srinivasan et al., 2015). Supporting these skills may increase the enjoyment of walking together and therefore increase the likelihood that the activity might be repeated more regularly.

### Communication

**Figure 6.**
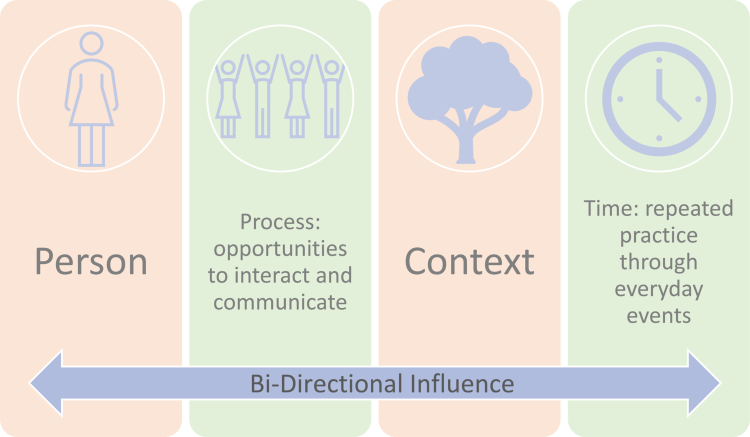
The process of communication over time.

Increased opportunities for communication were reported by parents of preschoolers with autism, who wrote about the ways their children pointed, commented or asked questions while walking. This is consistent with the literature which suggests that richer conversation may be observed in outdoor settings than indoors (Cameron-Faulkner et al., 2018; Novikova et al., 2024; Richardson et al., 2024). The combination of the setting, the outdoor natural environment, and the microsystem of parent and child, may lead to developmental growth through the regular process of conversation. Bronfenbrenner emphasised the importance of time, that is the influence of repeated events, especially everyday events, rather than one-off occurrences. Activities such as a daily walk in a comfortable setting may provide opportunities for regular practice in communication skills, including pointing, vocalisations, and verbal interactions (see Figure 6).

### Priority

**Figure 7.**
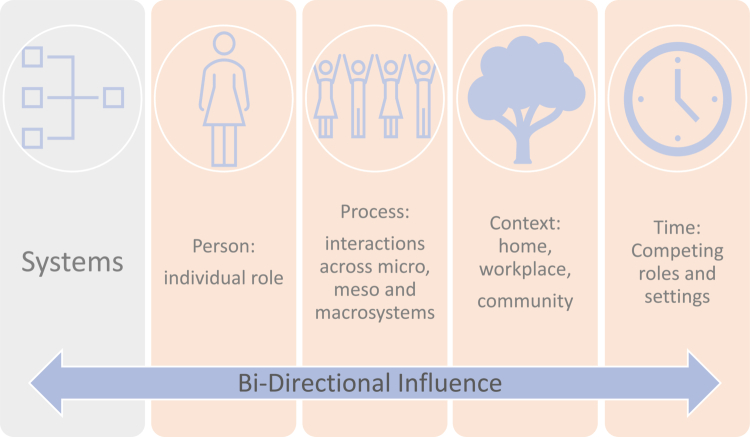
Change across systems.

Both parents with and without children with autism were asked to what extent walking in nature was a priority. Most of those responding agreed that it was, although some participants responded that other activities were prioritised over a walk in nature. Competing priorities including the needs of other children in the family, their need for therapy and preschool, and the effort needed to go outdoors mean that individual factors can affect whether walking outside is considered a priority at a particular point in time. Bronfenbrenner referred to the tension that might occur when individuals are expected to fulfil different roles simultaneously, with the example of a woman being expected by a husband to be a full-time mother and by an employer a full-time worker (Bronfenbrenner, 1979, *p*. 212). This conflict may compromise developmental growth. Altering a setting or process in one system, for example the microsystem, may not be enough to effect beneficial change; it is important that support for change occurs across different systems (see Figure 7). Allowing parents more flexibility with work, incorporating functional family goals into therapy, and providing practical help to complete tasks in the home may all be ways of decreasing conflicts which prevent walking in nature from being prioritised.

## Limitations

Limitations of this study were found in recruitment, where only participants who were fluent in English and computer-literate were able to respond to the survey. We were careful not to specify that the survey was about access to nature in the initial advertising; however, it is possible that many of the incomplete responses were from parents who were not interested in nature, or who had children who were overwhelmed or disliked natural settings. Other recruitment strategies which may have limited participants include the use of Facebook groups, meaning those who chose not to participate in social media were less likely to hear about the study. The ages and diagnoses of the children were not independently verified, which could lead to inaccuracies in participant perspectives. Future research should consider these issues when recruiting, although generalisations of the outcomes of any exploratory research should be limited.

## Conclusion

In this study, parents reflected on their experiences of walking with their preschooler with or without autism, either after a specific walk or on walking in general. Participants noted moments of social interaction such as holding hands and walking side by side, different types of communication attempts from questions to pointing, and physical interaction with the environment such as jumping, splashing and climbing. These experiences provided the opportunities for learning and growth that researchers suggest may occur in natural settings (Jimenez et al., 2021). However, some families found that competing needs and particular challenges associated with autism, including difficulties walking in synchrony, meant that while they valued a walk in nature, it was not considered a priority.

Our analysis makes visible the multiple, interacting systems which affect the way parents of preschool children with autism can partake in the commonplace activity of going for a walk in a natural setting. Some models, such as the medical model, focus entirely on the individual factors which affect development; other models, such as a social model, focus entirely on external attitudes within a community and society (Tregaskis, 2004). Bronfenbrenner’s bio-ecological framework, however, suggests a more complex system; that individual (including genetic) differences interact with relationships, settings, socio-cultural and legal realities, and that all these systems affect one another. Change can only happen when these multiple factors are accepted and accounted for.

Further research could extend these findings by recruiting families from other under researched groups, such as those unable to respond in English. An addition of ‘walking interviews’, where the researcher observes a family while walking, could complement parental perceptions (see Clement & Waitt, 2017). Experimental data comparing family functioning and developmental outcomes based on time in nature, nearness to natural settings, or the effect of nature prescriptions could also be fruitful. Walking in nature with family is an underrated activity with a wide range of benefits for adults and children, including those with autism.

## Data Availability

The data presented in this article are not readily available due to personal privacy restrictions.

## Endnotes

^1^
The authors acknowledge the diverse language preferences of the autism community, using both “children/adolescents with autism” and “autistic individuals/adults” in this article, as suggested by Bury et al. (2023) among others.
